# On the Birth of Journal of Neonatal Surgery

**Published:** 2012-01-01

**Authors:** Yogesh Kumar Sarin, Sushmita Bhatnagar

**Affiliations:** Department of Pediatric surgery, Maulana Azad Medical College New Delhi, INDIA; 1 Department of Paediatric Surgery BJ Wadia Hospital for Children Mumbai, INDIA

"Dreams are the seedlings of reality”- Napoleon Hil

The dream of Dr. M. Bilal Mirza became a reality on 17th July 2011 when his surreal idea of starting a new online Journal of Neonatal Surgery was endorsed by us. And now in less than six months, on the occasion of the New Year of 2012, with the concerted help and support of learned colleagues, we deliver the first issue of the Journal of Neonatal Surgery.

Neonatal surgery is the flagship and most challenging component of pediatric surgery; the quality of neonatal surgical service is a measure of the quality of pediatric surgical service provided by any centre. It is what separates us from adult general surgeons who may operate on children, but who do not have the expertise in treating newborn surgical problems.

Neonatal surgery encompasses not only the surgical details and techniques of correction of the congenital malformations, but also understanding of the neonatal physiology, the patho-physiology, the spectrum of anomalies, genetics, the diagnostic modalities and advances in technology and neonatal intensive care. In no other components of pediatric surgery, the pre-operative preparation and post-operative management is as critical for a successful outcome as in neonatal surgery. The era when surgical neonates used to be housed in one of the many cubicles at the end of children’s surgical ward is a thing of the past.

Nowadays, in the developed world, multidisciplinary approach has evolved where pediatric/neonatal surgeons work in tandem with the perinatologists, obstetricians, neonatologists, pediatric cardiologists, pediatric neurosurgeons, pediatric anesthesiologists to optimize the management and outcome in these patients. Such collaboration is very important in the case of preterm and term low birth weight babies, where temperature regulation, fluid balance, assessment of metabolic disturbances, respiratory function, management of nutrition etc are vital for a successful outcome. All this progress has not happened overnight. Surveys have been conducted to know the ground realities [1]. Neonatal surgical care modules and curricula have been developed, both for the trainee doctors as well as nurses [2]. New concepts like operating the surgical neonates in NICU itself have emerged [3, 4].

However, neonatal surgery is still bedeviled by a myriad of problems, making newborn surgery to be attended by unacceptable morbidity and mortality in the third world countries [5]. The problems range from lack of awareness and late presentation, through shortage of relevant personnel and lack of appropriate equipments and facilities to poor financing for neonatal surgical services. To improve the prevailing situation, efforts need to be made by all stakeholders, including the governmental and international agencies [6].

Through Journal of Neonatal Surgery, we intend to provide a portal to research and clinical material relating specifically to the surgical neonate. None of the existing six English journals of Pediatric Surgery devote even a small dedicated section to neonatal surgery. We believe that neonatal surgery has emerged a specialty in its own right over last two decades and should have a scientific journal devoted entirely to it. Besides the neonatal surgeons, all those caring for these unfortunate neonates, be the neonatologists, the neonatal nurses, the radiologists, the neonatal anesthetists or the geneticists could share their knowledge, expertise and research on a common platform. Although the first issue appears to be an isolated Indo-Pak venture, we are sure that soon the journal would have international reach and we shall receive valuable contributions from all parts of the globe.

## Footnotes

**Source of Support:** Nil

**Conflict of Interest:** None declared

